# 2149: Primary management of advanced-stage ovarian cancer: 1 year at a high-volume care center

**DOI:** 10.1017/cts.2017.116

**Published:** 2018-05-10

**Authors:** Maureen Byrne, Renee Cowan, Jennifer Spross, Kara Long-Roche, Ginger Gardner

**Affiliations:** Icahn School of Medicine at Mount Sinai, New York, NY, USA

## Abstract

OBJECTIVES/SPECIFIC AIMS: To describe the use of primary debulking surgery and neoadjuvant chemotherapy in advanced-stage ovarian cancer patients treated at Memorial Sloan Kettering Cancer Center (MSKCC) over the period of 1 year. Specifically, identify a subset of patients that are medically eligible to be considered for surgery. Examine the ultimate treatment designation for those patients, assessing the application of the MSKCC resectability algorithm and its utility in guiding treatment choice. METHODS/STUDY POPULATION: Using the prospectively maintained Ovarian Cancer Database at MSKCC, we queried patients who presented for initial management of ovarian cancer from July 1, 2015 to June 30, 2016. All patients with stage IIIB-IV disease who received their primary treatment at MSKCC were included in our study. Patients needed to have pathology-confirmed ovarian cancer and all histological subtypes were included. Data were collected and analyzed in Excel. RESULTS/ANTICIPATED RESULTS: There were a total of 173 patients treated for stage IIIB-IVB ovarian cancer at MSKCC during the study period. Of those 98 patients received PDS, whereas 75 were directed to NACT, making MSKCC’s overall NACT rate 43.4% for the year we studied. Of the patients who received NACT, 19 met full Aletti Criteria at diagnosis, precluding them from being considered for surgery. In addition, 21 patients had medical contraindications to surgery, meaning that a total of 40 patients who were given NACT were not able to be considered for PDS. If we then take into account only the patients who were medically eligible for PDS, the rate of NACT at MSKCC drops to 23.1%, almost half of the original value. These medically eligible patients are the population that should be receiving an MSKCC resectability score. Of the 98 patients who underwent PDS, 73.5% had a preoperative resectability score calculated. Based on the algorithm, 81.3% of those patients were deemed to be “low risk” and 15.2% were deemed to be “high risk” of a suboptimal debulking. The algorithm dictates that all “high risk” patients who go on to PDS should undergo a laparoscopy first to assess for resectability and potentially avoid an unnecessary open procedure. Hundered percent of the “high risk” cases that were taken to the OR had an initial laparoscopy before proceeding with PDS. Overall, 93.1% of patients that underwent PDS had an optimal cytoreduction, or ≤1 cm residual disease at the conclusion of surgery. Of the 6 patients throughout the year that had a suboptimal outcome, or >1 cm residual disease, 3 were initially scored as “low risk,” 1 was scored as “high risk,” and 2 did not receive an MSKCC resectability score prior to their procedure. Of note, 3 of the suboptimal cases had unresectable disease in an anatomical location not accounted for in the resectability algorithm. DISCUSSION/SIGNIFICANCE OF IMPACT: The rates of PDS Versus NACT vary widely between institutions, and it is not always clear how calculations are made. High-volume centers likely see a higher percentage of sicker patients with more advanced disease, which could increase their rates of NACT as many of these patients are not eligible for surgery. It is important to standardize the way our field quotes NACT rates, and to understand how treatment decisions are being made at a given institution. PDS has a demonstrated survival benefit, and while we would ideally use this modality for all of our patients, there will always be a baseline percentage of patients who cannot be considered for the surgery. Since we will never be able to offer those patients PDS, our objective should be to identify patients who can be considered for the procedure and to work toward optimizing their outcomes. In this study we identified the population of patients who are truly the PDS Versus NACT cohort as they were eligible for both modalities. We then examined the application and utility of the MSKCC resectability algorithm in an attempt to further optimize treatment allocation. This scoring system was implemented at our hospital over the past year with the goal that 100% patients going on to PDS would receive a preoperative score. Unfortunately, 26.5% of PDS patients were not scored prior to their procedure. This makes it more difficult to evaluate the efficacy of the scoring system, especially considering 1/3 of the suboptimal cases were not scored. Had these patients received a score, they might have been deemed “high risk” and could have avoided a lengthy operation with a significant chance of a suboptimal outcome. In addition, it is important to note that 3 of the suboptimal PDS outcomes were initially scored as “low risk,” and 3 of the suboptimal outcomes were due to disease locations not accounted for in the original resectability algorithm. We will continue logging disease locations of suboptimal cases, it is possible that a certain disease location not in the scoring system is responsible for a significant portion of suboptimal outcomes. The resectability score model had an overall predictive accuracy of 0.756 when it was initially published, and we must continue tracking scores and outcomes to determine its validity when applied prospectively in our population. In order to accurately do so however, an emphasis should be made to ensure 100% of patients being considered for PDS receive a score going forward.

